# Prevalence and transmission characteristics of *Listeria* species from ruminants in farm and slaughtering environments in China

**DOI:** 10.1080/22221751.2021.1888658

**Published:** 2021-03-01

**Authors:** Qiang Zhao, Pan Hu, Qianqian Li, Shasha Zhang, Hanxiao Li, Jiang Chang, Qiujie Jiang, Yu Zheng, Yansong Li, Zengshan Liu, Honglin Ren, Shiying Lu

**Affiliations:** a Key Laboratory of Zoonosis Research, Ministry of Education, Institute of Zoonosis, Double First-class Discipline of Human-animal Medicine, Jilin University, Changchun, People’s Republic of China; b Jilin Center for Animal Disease Control and Prevention, Changchun, Jilin, People’s Republic of China

**Keywords:** *Listeria monocytogenes*, *Listeria innocua*, cattle and sheep, farm, slaughtering, raw milk, transmission characteristics

## Abstract

*Listeria monocytogenes* is an important foodborne pathogen, and is ubiquitously distributed in the natural environment. Cattle and sheep, as natural hosts, can transmit *L. monocytogenes* to related meat and dairy products. In this study, the prevalence, distribution, and transmission characteristics of *Listeria* were analysed by investigating 5214 samples of cattle and sheep in farm and slaughtering environments in China. A low contamination incidence of *L. monocytogenes* (0.5%, 20/4430) was observed in farm environment, but there was a high contamination incidence in slaughtering environment (9.4%, 74/784). The incidence of *L. innocua* in cattle and sheep farm and slaughtering environments is more common and significantly higher (9.7%, 508/5214) than that of *L. monocytogenes* (1.8%, 94/5214). The distinct molecular and genetic characteristics of *Listeria* by PFGE and MLST indicated that *L. monocytogenes* and *L. innocua* were gradually transmitted from the farm and slaughtering environments to end products, such as beef and mutton along the slaughtering chain. The ST7, ST9, ST91, and ST155 found in our study were associated with the human listeriosis cases in China. In addition, the findings of virulence markers (*inlC*, *inlJ*, LIPI-3*,* LIPI-4, and ECIII) concerned with the pathogenesis of human listeriosis and antibiotics resistance of *L. monocytogenes* in this study implies a potential public health risk. This study fills the gap in the epidemiology of beef cattle and sheep that carry *Listeria* in farm and slaughtering environments in major cattle and sheep producing areas in China.

## Introduction

*Listeria* spp. are widely distributed in various environments due to its adaptability to various harsh conditions. It has been isolated from soil, water, plants, faeces, rotten vegetables, fruits, meat, seafood, dairy products, and asymptomatic carriers of human and animals [1]. By 2020, there are currently 21 species had been identified: *L. aquatica, L. booriae, L. cornellensis, L. costaricensis, L. fleischmannii, L. floridensis, L. goaensis, L. grandensis, L. grayi, L. innocua, L. ivanovii, L. marthii, L. monocytogenes, L. newyorkensis, L. riparia, L. rocourtiae, L. seeligeri, L. thailandensis, L. valentina, L. weihenstephanensis,* and *L. welshimeri* [2].

*L. monocytogenes* is the main pathogen of human listeriosis, and an important foodborne pathogen. It is characterized by low incidence rate and high mortality, and can cause serious damage to pregnant women, newborn infants, the elderly, and the immunocompromised population [3]. It has the capacity to grow slowly at 4°C in the refrigerator, posing potential threat to the health of general public. Lots of researches have been done to detect *L. monocytogenes* persisting in meat products, vegetables, milk, frozen food, etc. in China [4–10]. In addition, other *Listeria* spp. except *L. monocytogenes* were also proved to be virulent in recent years [11,12]. *L. ivanovii* was reported to cause diseases mainly in mammals. Recent years, *L. innocua* is divided into two kinds: typical *L. innocua* and atypical haemolytic *L. innocua*, the former is thought to be non-haemolytic, and the latter is thought to be virulent, albeit less than *L. monocytogenes* [12].

Many animals are natural hosts of *Listeria* spp., including cattle and sheep, which are natural repositories of *Listeria*. *L. monocytogenes* was reported to isolate from clinically infected and clinically normal cows on dairy farms, which can frequently shed *Listeria* spp. into faeces through the intestinal track, disseminating the pathogen into the farm environment [13]. There have been many reports that *L. monocytogenes* will cause widespread cross-contamination of final animal-derived products. The hides and intestines are thought to be the most important sources for microbial contamination [14]. Therefore, the investigation of livestock animals in farm environment is very important to elucidate the contaminant source of *L. monocytogenes*, which will provide crucial data for control of *L. monocytogenes* at the farm level [15].

The output of beef and mutton in China ranks first in the world, and the demand and proportion of meat are increasing year by year. However, the prevalence and transmission background of *L. monocytogenes* in cattle and sheep farm environments, and meat and dairy products in major cattle and sheep producing areas in China remain unclear. At present, the surveillance data of *Listeria* distribution in farm and slaughtering environments of cattle and sheep remain limited in China. Thus, it has become difficult for policymakers to formulate effective policies to control the *Listeria* contamination in food of cattle and sheep origin. This study was conducted to reveal the prevalence, transmission, and pheno- and genotypic characteristics of *Listeria* species in farm and slaughtering environments in major cattle and sheep producing areas in China.

## Materials and methods

### Sample collection

**Figure 1.**
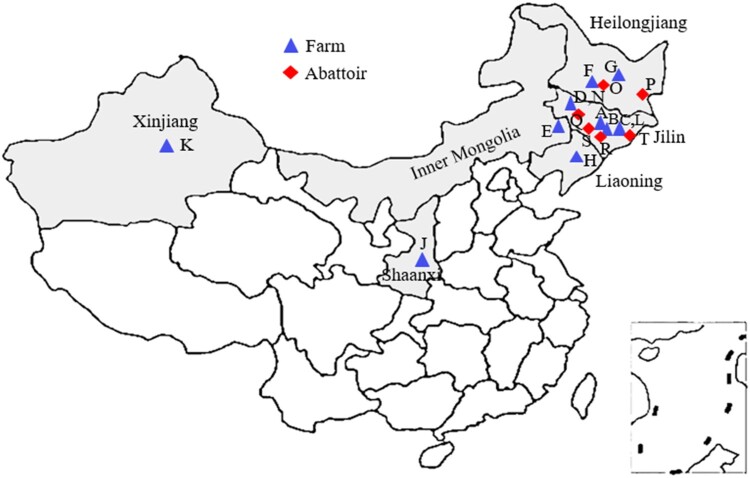
The geographic locations of samples from farm and slaughtering environments. The triangular shades of blue represent the different farms of beef cattle, dairy cattle or sheep, and shades of red prismatic stand for different abattoirs of beef cattle, or sheep in major cattle and sheep producing areas in China. (A) Dairy cattle farm located in Changchun; (B) beef cattle farm located in Yitong; (C) beef cattle farm located in Dunhua; (D) beef cattle farm located in Zhenlai; (E) beef cattle farm located in Tongliao; (F) beef cattle farm located in Daqing; (G) dairy cattle farm located in Suihua; (H) dairy cattle farm located in Shenyang; (J) beef cattle farm located in Shaanxi; (K) beef cattle farm located in Xinjiang; (L) sheep farm located in Dunhua; (N) sheep farm located in Zhenlai; (O) beef cattle abattoir in Daqing; (P) beef cattle abattoir in Jixi; (Q) sheep abattoir in Zhenlai; (R) beef cattle abattoir in Siping; (S) beef cattle abattoir in Changchun; (T) beef cattle abattoir in Yanji.

Faeces, silage, drinking water, hide swabs, and raw milk samples directly from the nipple of dairy cattle in milk halls in farm environment, and faeces, hide swabs, knife swabs, rinsed water used to clean the knife, chopping board swabs, instrument swabs, and beef for sale after slaughter in the slaughtering environment were gathered in major cattle and sheep producing areas in China from 1 September 2018 to 1 October 2019. A total of 5214 samples were randomly collected from farms and abattoirs located in Northeast China, Northwest China, and Inner Mongolia (Figure 1), covering 12 farms and 6 abattoirs in six provinces in China.

### Isolation, PCR and serological identification of *Listeria* spp.

*Listeria* were isolated according to the GB 4789.30–2010 method in China, with moderate modifications [16]. Ten millilitres *Listeria* Enrichment Broth Base LB_1_ (Haibo Biology, Qingdao, China) were prepared before the sampling was carried out each time. Approximately 2 g, 2 mL, or 2 cm^2^ of samples were collected and directly added into 10 mL of LB_1_ once samples were gathered in farms or abattoirs. Then, 25 g of beef or mutton samples and 225 mL of LB_1_ were used for immediate homogenizing when these were transported to the laboratory. After the samples were incubated at 30°C for 24 h, 0.1 mL of preliminary enrichment broth was added into 10 mL of LB_2_ for the second-step enrichment at 30°C for 24 h. Then, two loopful of secondary enrichment broth were inoculated on PALCAM Agar (Haibo Biology, Qingdao, China) at 30°C for 28 h. Afterwards, 3–5 presumptive colonies were randomly chosen for purification and subculture on the PALCAM agar at 37°C for 24 h. Then, the purified bacterial cultures were chosen for Gram-staining. After Gram-staining, cultures suspected of being *Listeria* spp. were further checked using the multiplex PCR method that targets specific genes of different *Listeria* spp. [17]. Primers targeting the putative transcriptional regulator gene were used to check the *L. innocua* strains [18]. In addition, a multiplex PCR method to differentiate *L. monocytogenes*, *L. ivanovii*, typical *L. innocua*, and atypical *L. innocua* was used to check the above results again [11]. The *Listeria* strains were preserved in LB liquid medium that contained 20% glycerol at −80°C.

The bacterial wall-breaking treatment and multiplex PCR method to differentiate the major serogroups of *L. monocytogenes* were applied, according to the description reported by Doumith et al. [19]. When the serogroups were identified, traditional slide agglutination with the *Listeria* Antisera (Denka Seiken, Japan) and polyclonal crossed absorbed factor (*L. innocua*: 6a, 6b) antiserum, in accordance with the methods described by Seeliger and Hönne [20], which were preserved by our laboratory, was operated according to the instructions of the manufacturer, in order to identify the specific serotype of *Listeria isolates*.

### Pulsed-field gel electrophoresis

The pulsed-field gel electrophoresis (PFGE) of the strains on 57 representative *Listeria* strains based on different regions, types of samples, and serotypes was performed using the primary restriction enzyme *Apa*I (Takara, Dalian, China), according to the standard operating procedure provided by the PulseNet of Centers for Disease Control and Prevention [21]. Similarities among the fingerprint profiles of strains were analysed by the unweighted pair group method with arithmetic mean using the BioNumerics software (Version 5.10, Applied Maths, Belgium). The *Salmonella* strain H9812 restricted with *Apa*I was used for molecular weight standards in all the PFGE gels.

### Multi-locus sequence typing

Multi-locus sequence typing (MLST) on 50 *L. monocytogenes* and 50 *L. innocua* isolates from different regions based on seven house-keeping genes (*abcZ*, *bglA*, *cat*, *dapE*, *dat*, *ldh*, and *lhkA*) was performed, according to the primers and the methods provided in http://bigsdb.web.pasteur.fr/Listeria/. The sequences types (STs) were determined by comparing with the allelic profiles for *Listeria* in the MLST database. The minimum spanning tree construction based on the seven house-keeping genes’ sequences was analysed using the BioNumerics software.

### Detection of virulence markers of *L. monocytogenes*

Virulence genes *inlC* and *inlJ* were detected simultaneously using PCR methods described by Liu et al. [22]. The presence of LIPI-3 encoding listeriolysin S and LIPI-4 encoding a cellobiose-family phosphotransferase system were confirmed by using PCR targeting *llsX* and *ptsA* genes, respectively, to screen for the potential hypervirulent *L. monocytogenes* [8,23]. ECIII associated with outbreaks in the United States was also determined for 1/2a isolates by using PCR [24]. In addition, mutations leading to a premature stop codon (PMSC) in *inlA* that significantly reduce the virulence of *L. monocytogenes* were analysed [8].

### Antimicrobial susceptibility test

Antimicrobial susceptibility test of all *L. monocytogenes* strains was done using the disk diffusion method according to the Clinical and Laboratory Standard Institute (CLSI) guidelines [25]. A total of 18 antibiotic agents were tested at specific concentrations per disk (Table 4). *Staphylococcus aureus* ATCC 25923 and *Escherichia coli* ATCC 25922 were used as quality control strains. Strains with three or more antibiotic resistance were defined as multidrug-resistant strains [26].

### Statistical analysis

The chi-square test was used to determine statistical differences of the prevalence of *Listeria* spp. among the farms, abattoirs, and sample categories. *P *<* *.01 was considered as having a highly significant difference, while *P *<* *.05 was regarded as having a significant difference. All statistical analyses were performed using the SPSS v25.0 software.

## Results

### Occurrence of *Listeria* spp. in farm and slaughtering environments of ruminants

The incidences of *Listeria* spp. in various samples or different farms and abattoirs are summarized in Tables 1 and 2, and Tables S1–S3. The prevalence rate of *Listeria* spp. in farm environment varied from 0% to 18.4% among different farms. Among the samples of different categories in farm environment, the prevalence rates of *Listeria* spp. varied within 1.9–7.8%. Furthermore, the prevalence rates of *Listeria* spp. varied from 22.9% to 69.5% among different abattoirs. Among different categories of samples in slaughtering environment, the prevalence rates of *Listeria* spp. varied within 17.4–60.0%. The incidence of *Listeria* spp. in farm F in Daqing or farm H in Shenyang was highly significant, when compared with that in the other farms (*P *<.01). However, the difference of these between farm F and farm H was not statistically significant (*P *> .05). The incidence of *Listeria* spp. in abattoir S in Changchun exhibited highly significant difference, when compared with that in other abattoirs (*P *<.01). The incidence of *Listeria* spp. in faeces or silage was highly significant than in the other sample categories in farm environment. There were no statistically significant differences between faeces and silage (*P *> .05). The incidence of *Listeria* spp. in beef was highly significant than in the other sample categories expect rinsed water and chopping board in slaughtering environment (*P *<.01). There were no statistically significant differences among beef, rinsed water, and chopping board (*P *> .05).

**Table 1.** Summary of data on the prevalence of *Listeria* according to regions and sample categories.

	Number of sample	M^a^ (%)	I^a^ (%)	*Listeria* (%)
**Feeding environment**				
Faeces	3284	8 (0.2)	188 (5.7)	196 (6.0)
Hide swab	320	0 (0)	6 (1.9)	6 (1.9)
Silage	204	2 (1.0)	14 (6.9)	16 (7.8)
Drinking water	131	1 (0.8)	4 (3.1)	5 (3.8)
Raw milk	491	9 (1.8)	2 (0.4)	11 (2.2)
Total	4430	20 (0.5)	214 (4.8)	234 (5.3)
**Slaughtering environment**				
Anal swab**^b^**	121	10 (1.0)	11 (23.1)	21 (17.4)
Fur swab	76	8 (10.5)	21 (27.6)	29 (38.2)
Rinse water	23	0 (0)	12 (52.2)	12 (52.2)
Knife	64	8 (12.5)	17 (26.6)	25 (39.1)
Instrument	30	0 (0)	10 (33.3)	10 (33.3)
Chopping board	32	1 (3.1)	15 (46.9)	16 (50)
Carcass	71	11 (15.5)	17 (23.9)	28 (39.4)
meat	367	36 (9.8)	191 (5.2)	220 (60.0)^c^
Total	784	74 (9.4)	294 (37.5)	361 (46.0)

^a^ M, *L. monocytogenes*; I, *L. innocua*.

^b^ Farm environment related with cattle in abattoir.

^c^ *L. monocytogenes* and *L. innocua* coexisted in 7 beef samples. Therefore, the total number of *Listeria*-positive samples was not 227, but was 220.

**Table 2.** Summary of data on the prevalence of *Listeria* according to regions.

	Number of sample	M^a^ (%)	I^a^ (%)	*Listeria* (%)
**Farm environment**				
A	637	9 (1.4)	1 (0.2)	10 (1.6)
B	150	0 (0)	0 (0)	0 (0)
C	206	0 (0)	19 (9.2)	19 (9.2)
D	100	0 (0)	0 (0)	0 (0)
E	400	0 (0)	0 (0)	0 (0)
F	463	0 (0)	85 (18.4)	85 (18.4)
G	1264	0 (0)	28 (2.4)	28 (2.4)
H	470	10 (2.1)	71 (15.1)	81 (17.2)
J	130	0 (0)	0 (0)	0 (0)
K	160	0 (0)	0 (0)	0 (0)
L	250	1 (0.4)	10 (4)	11 (4.4)
N	200	0 (0)	0 (0)	0 (0)
Total	4430	20 (0.5)	214 (4.8)	234 (5.3)
**Slaughtering environment**				
O	287	59 (20.6)	82 (28.6)	141 (49.1)
*P*	80	0 (0)	40 (50)	40 (50)
Q	70	0 (0)	18 (25.7)	18 (25.7)
R	20	0 (0)	6 (30.0)	6 (30.0)
S	174	10 (5.7)	118 (67.8)	121 (69.5)^b^
T	153	5 (3.3)	30 (19.6)	35 (22.9)
Total	784	74 (9.4)	294 (37.5)	361 (46.0)

^a^ M, *L. monocytogenes*; I, *L. innocua*.

^b^ *L. monocytogenes* and *L. innocua* coexisted in seven beef samples. Therefore, the total number of *Listeria*-positive samples was not 128, but was 121.

A total of 1025 *Listeria* strains were isolated from 5214 samples, in which the amount of isolated *L. monocytogenes* strains was 117 (11.41%) and the amount of isolated *L. innocua* strains was 907 (88.49%). Merely one atypical *L. innocua* strain was isolated from farm H, while the remaining *L. innocua* strains were all typical ones. All *L. monocytogenes* strains were divided into 1/2a (70.09%) and 1/2c (29.91%) serotypes by multiplex PCR and *Listeria* antiserum.

During the process of species and serological identification, it was observed that 1–3 serotypes of *Listeria* (*L. monocytogenes*: 1/2a, 1/2c; *L. innocua*: 6a, 6b) from the same samples were isolated.

### Pulsed-field gel electrophoresis

**Figure 2.**
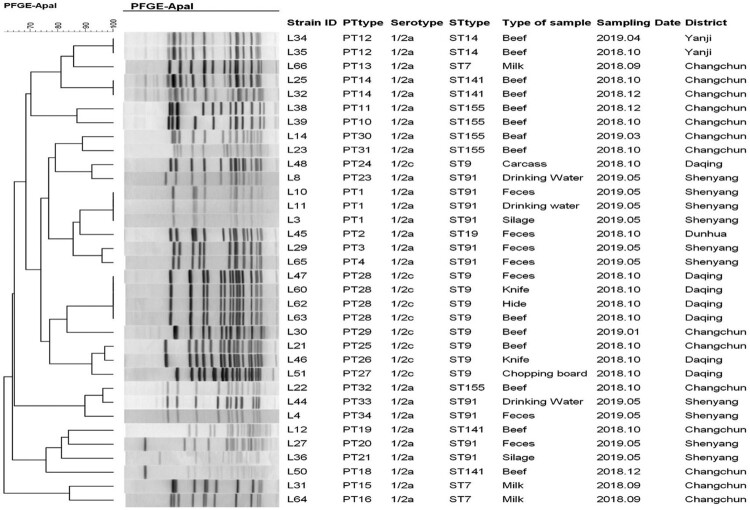
The dendrogram generated by the *Apa*I enzyme-based pulsed-field gel electrophoresis patterns of the 34 representative *L. monocytogenes* strains was constructed. The corresponding data, including the name of the strain (Strain ID), PFGE types, serotype, MLST type, the type of sample, sampling date and district, are shown alongside the dendrogram to the right.

The PFGE analysis with the *Apa*I enzyme divided the 34 *L. monocytogenes* strains into 27 pulsotypes (PTs) (Figure 2), and 23 *L. innocua* strains into 17 PTs (Figure S1), with diverse clusters. Identical *L. monocytogenes* strains with same serotypes, PTs and STs from different samples were observed only in the same region. The 11 *L. monocytogenes* strains (32.4%) exhibited six kinds of identical PTs and STs. Identical *L. monocytogenes* isolated from cattle and sheep farm and slaughtering environments had regional correlation. The strains which were identical among faeces, silage, and drinking water in farm environment, and the identical strains among slaughtering environment, intestinal tract, and beef were found. The *Listeria* strains from the same abattoir had genetic correlation in time and space. PFGE showed that there were transmission correlation and genetic association of *Listeria* in cattle and sheep farm, slaughtering environment and meat chain, which had a typical continuous transmission chain.

### Multi-locus sequence typing

**Figure 3.**
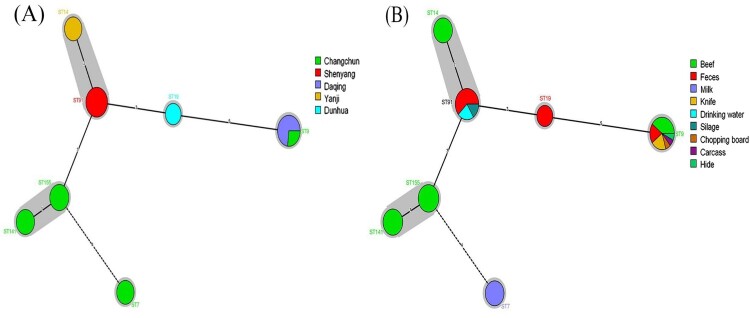
The minimum spanning tree of the seven STs of the 50 *L. monocytogenes* isolates obtained from ruminants in farm and slaughtering environments in China. The corresponding sequence type is displayed around the circles. The size of each circle corresponds to the isolate count, and the colour within these circles represent the type of region (A) or sample (B).

A total of 24 different STs were classified among all 100 *Listeria* spp. isolates, which were further assigned to 23 clonal complexes (CCs): 50 *L. monocytogenes* strains were divided into 7 STs (6 CCs) and 50 *L. innocua* were divided into 17 STs (17 CCs). The MLST data revealed that the most prevalent STs of *L. monocytogenes* were ST9 (9 strains, 36%) and ST91 (8 strains, 32%). The minimum spanning tree of *Listeria* isolates is shown in Figure 3 and Figure S2, presenting the genetic correlation among strains of different regions or categories of samples.

### Detection of virulence markers

The results of virulence markers of 117 *L. monocytogenes* are shown in Table 3. All strains carried the *inlC* and *inlJ* genes (100%). Four LIPI-3 and LIPI-4-positive isolates (belonged to 1/2a/ST91 and 1/2c/ST9) were found in farm H and abattoir O and four isolates that were only positive for LIPI-4 (belonged to 1/2a/ST141) were found in abattoir S. In addition, three isolates in abattoir T were found to be ECIII (belonged to 1/2a/ST14). Eighteen *L. monocytogenes* isolates contained PMSCs, including mutation type 4 (14/18, existed in 1/2a/ST7, 1/2c/ST9, 1/2a/ST91 and 1/2a/ST155), type 8 (3/18, existed in 1/2c/ST9), and type 12 (1/18, existed in 1/2c/ST9). LIPI-3/LIPI-4, ECIII, and PMSC in *inlA* existed independently and did not intersect with each other.

**Table 3.** Detection results of virulence markers.

Environment	No. of strains	*inlC* (%)	*inlJ* (%)	LIPI-3(%)	LIPI-4 (%)	ECIII (%)	PMSC in *inlA* (%)
Farm	45	45 (100)	45 (100)	1 (2.2)	1 (2.2)	0 (0)	8 (17.8)
Abattoir	72	72 (100)	72 (100)	3 (4.2)	7 (9.7)	3 (4.2)	10 (13.9)
Total	117	117 (100)	117 (100)	4 (3.4)	8 (6.8)	3 (2.6)	18 (15.4)

### Antimicrobial susceptibility test

The antimicrobial resistance analysis of 18 antibiotic agents against the 117 *L. monocytogenes* isolates is shown in Table 4. All strains were susceptible to two antibiotics, penicillin and imipenem. The most frequent antibiotic resistance was to clindamycin (71.8%), streptomycin (71.8%), amikacin (65.8%), and cefuroxime (61.5%). Notably, three strains belonged to ECIII were resistant to 10 antibiotics.

**Table 4.** Antibiotic resistances of *L. monocytogenes* isolates.

Antibiotics (disk content, μg or U)	No. of isolates (%)		
	Susceptible	Immediate	Resistance
Penicillin G (10 U)	110 (94.0)	0 (0)	7 (6.0)
Ampicillin (10)	77 (65.8)	20 (17.1)	20 (17.1)
Cefuroxime (30)	25(21.4)	20 (17.1)	72 (61.5)
Amikacin (30)	7 (6.0)	33 (28.2)	77 (65.8)
Gentamcin (10)	39 (33.3)	26 (22.2)	52 (44.4)
Kanamycin (30)	7 (6.0)	52 (44.4)	58 (49.6)
Streptomycin (10)	0 (0)	33 (28.2)	84 (71.8)
Tetracycline (30)	39 (33.3)	26 (22.2)	52 (44.4)
Erythromycin (15)	45 (38.5)	52 (44.4)	20 (17.1)
Ciprofloxacin (5)	0 (0)	104 (88.9)	13 (11.1)
Vancomycin (30)	84 (71.8)	20 (17.1)	13 (11.1)
Novobiocin (30)	90 (76.9)	7 (6.0)	20 (17.1)
Trimethoprim-sulphamethoxazole (1.25/23.75)	97 (82.9)	0 (0)	20 (17.1)
Amoxicillin (20)	117 (100)	0 (0)	0 (0)
Rifampin (5)	46 (39.3)	38 (32.5)	33 (28.2)
Chloramphenicol (30)	72 (61.5)	13 (11.1)	32 (27.4)
Clindamycin (2)	7 (6.0)	26 (22.2)	84 (71.8)
Imipenem (10)	117 (100)	0 (0)	0 (0)

## Discussion

There have been a few reports on the prevalence and distribution of *Listeria* spp. in food processing environments and retail food markets in China [27–31]. However, the prevalence and distribution of *Listeria* spp. from ruminants in farm and slaughtering environments have been rarely reported in China at present.

In this study, *L. monocytogenes* mainly presented in faeces samples (0.2%) in the farm environment, and the incidence of *L. monocytogenes* was 0.5% (20/4430). A higher prevalence rate of *L. monocytogenes* in the farm environment was reported in 1738 faeces samples of black beef cattle (6%) in Japan [15], and in 734 faeces samples of dairy cattle (43%) in New York State [32]. The prevalence of *L. monocytogenes* in raw milk samples produced by dairy cattle was 1.8% (9/441), which was lower than that reported by Kalorey et al. [33], which was 5.1% of the 2060 raw milk samples in Central India. The occurrence of *Listeria* spp. in raw milk samples may be associated with faeces, silage, and milking hygiene [34].

*L. monocytogenes* was found in 3.4% of bovine carcass in Turkey [35] and in 2.5% of bovine carcass in Poland [36]. The prevalence of which was lower than that in the present results for bovine carcass (19.6%) in the slaughtering environment. The incidence of *L. monocytogenes* and *Listeria* spp. in the farm environment (0.5% and 5.3%, respectively) was much lower than that in the slaughtering environment (9.4% and 46.0%, respectively). The incidence of *L. innocua* in cattle and sheep farm and slaughtering environments is more common and significantly higher (9.7%, 508/5214) than that of *L. monocytogenes* (1.8%, 94/5214).

The 1/2a, 1/2b, 1/2c, and 4b are the dominant serotypes for food strains in China, in which serotype 1/2a, 1/2b, and 4b accounted for most of the human clinical cases [3,37]. In addition, 1/2c was also found in human listeriosis in China [38]. In our study, merely strains of 1/2a and 1/2c serotypes were isolated in farm and slaughtering environments of cattle and sheep. This is consistent with the results, in which only the 1/2a and 1/2c serotypes were isolated by Zhu et al., Takashi et al. and Wieczorek et al. [36,39,40].

It is noteworthy that 1–3 *Listeria* strains of different serotypes in the same sample were isolated during the isolation and identification process of samples. In general, strains with one serotype were encountered in most samples. However, strains with 2–3 serotypes of *Listeria* in some samples were isolated in this study. Different species of the *Listeria* genus or different serotypes of one species in one sample should be considered as discrepant strains. It is suggested that at least five suspicious colonies should be selected from each sample in the process of isolating strains, when possible, in order to avoid the possibility of missing detection, and provide more comprehensive and objective data for large-scale laboratory and epidemiological investigation, instead of selecting only one viable colony for further identification.

The simple data for the incidence of *Listeria* spp. in the diagrams limitedly revealed the transmission characteristics. The objective transmission characteristics must be in conjunction with the epi­demiologic, laboratory, and environmental investigations. *Listeria* has an obvious regional epidemic in the cattle and sheep farm environment, in which the prevalence of *Listeria* in cattle and sheep farm environments is very low and *L. monocytogenes* was isolated only from Shenyang H farm and Dunhua L farm. It was surprising to note that the samples collected in farm B, D, E, J, K, and N in the breeding environment were negative for *L. monocytogenes* and other *Listeria* spp. In Inner Mongolia (Farm E) and Zhenlai (Farm D and farm N), the land was full of desertified soil, and the local annual rainfall was pretty less than the east region, where the silage was more likely to be completely dry. Furthermore, there was also no *Listeria* strains collected from Shaanxi (Farm J) and Xinjiang (Farm K) in northwest China, where there was yearly drought. Interestingly, *Listeria* spp. could be isolated in samples collected from all abattoirs including abattoir S in Changchun and abattoir T in Yanji in different batches (Tables S4 and S5) in the slaughtering environment, where the surrounding environment was moist, indicating that *Listeria* spp. might be the resident flora in the slaughtering environment in China and the moist environment is crucial for the survival of *Listeria*.

Although *L. monocytogenes* was not collected in some abattoirs, and was only isolated to *L. innocua*, from the *L. monocytogenes* isolation rate of abattoirs, the slaughtering environment is the key to control the risk of transmission of *Listeria* during the process of farm and slaughtering. In sharing a mutual growth environment, other *Listeria* spp. have a similar capacity to survive under harsh environments. The presence of *L. monocytogenes* could be masked by the presence of other *Listeria* spp., in particular, *L. innocua* and *L. ivanovii*, according to ISO 11290-1:2017. However, the general trend of the isolation, in which *L. innocua* accounts for the large proportion, remains incontrovertible. In the toxicity evaluation study conducted by the investigators on mice, some *L. innocua* strains were less virulent than *L. monocytogenes*. However, they also had some lethal toxicity. There may be a horizontal transfer of virulence genes in *Listeria* spp., which post a potential threat to public health [41,42]. In another additional research, the plate counting method was performed with all original samples in four abattoirs. The pollution level in these samples varied within 0–10^5^ CFU/g in beef samples (Table S6), suggesting that beef or mutton can be contaminated to high levels by *Listeria* spp. at the end of the slaughtering process.

In the food chain of beef and mutton, there is a characteristic pollution transmission chain between farm and slaughtering, which ultimately poses a greater risk to the safety of beef and mutton. In ruminants, carrying *L. monocytogenes* were linked to poorly fermented silage, and this might make ruminants directly exposed to *L. monocytogenes*. Identical strains (1/2a, PT1, and ST91) in the silage, water, and faeces collected from farm H showed an evident epidemiological association, indicating that *L. monocytogenes* was very likely to enter faeces through silage or drinking water. Animals in the farm environment were considered to take the role as a reservoir or amplifier. *Listeria* spp. enters into the faeces, which would contaminate the farm environment, resulting to the carrying of *L. monocytogenes* in the hides. Identical strains (1/2c, PT36, and ST9) in the faeces, hides, knives, and beef obtained from the same slaughtering environment indicated that the organism in faeces and hides can transmit to beef along knives. This suggests that *L. monocytogenes* can be transmitted from the farm environment to the slaughtering environment and meat products, and the slaughtering process plays a crucial role. Strains isolated from the same abattoir S in Changchun at two months apart and strains from abattoir T in Yanji at six months apart were also identical, suggesting that the same population of *L. monocytogenes* can persist in the same environment for a long period of time. The study on the transmission of *L. innocua* in the farm and slaughtering environments has been rarely elucidated, which is similar to *L. monocytogenes* in our research. Control measures must rely on rigorous and hygiene slaughter processing procedures before beef or mutton can be distributed to retail markets.

The ST7, ST9, ST91, and ST155 detected in our study were associated with human listeriosis cases in China [38]. The virulence markers detected in this study (*inlC*, *inlJ*, LIPI-3, LIPI-4, ECIII, and PMSC in *inlA*) were also closely related to *L. monocytogenes* in the pathogenesis of human listeriosis [43]. Hypervirulent CC4 strains carrying both *llsX* and *ptsA* are known to be overrepresented in human isolates [4]. In our research, a small number of isolates belonged to ST9/CC9, ST91/CC14, and ST141/CC155 were newly found to harboured *llsX* and/or *ptsA*, suggesting that they may pose a hyper-pathogenic risk to public health. Those LIPI-3 and/or LIPI-4-positive isolates belonged to lineage II, which was inconsistent with the finding that LIPI-3 was identified exclusively in a subset of lineage I [44]. It is probably because strains in farm or slaughtering environment have evolved these two genes through gene transfer under natural environmental pressures. The discovery of virulence markers and natural multidrug-resistance in *L. monocytogenes* indicates a potential public health risk and the need for continuous monitoring of the potential virulence of the bacteria and guidance on the proper use of antibiotics at the farm level.

In conclusion, this study is the first to investigate the prevalence, distribution, and transmission characteristics of *Listeria* spp. in large scale in the chain of cattle and sheep farm and slaughtering environments in China. This study provides strong evidence for the prevalence and transmission characteristics of *Listeria* species in cattle and sheep farm and slaughtering environments, which include the milk production link. *L. monocytogenes* can be transmitted from the farm environment to the slaughtering environment and end meat products. The pathogenic genotypic characteristics and antibiotic resistance phenotypes of *L. monocytogenes* implies a potential public health risk*.* This study fills the epidemiological gap of *Listeria* species carried by cattle and sheep in farm and slaughtering environments in China, and provides a scientific basis for the prevention and control of listeriosis in humans and animals.

## Supplementary Material

Supplemental_material_for_review_.docx
