# Supplementary material for: Prevalence and transmission characteristics of Listeria species from ruminants in farm and slaughtering environments in China
Source: Emerg Microbes Infect. 2021 Mar 1;10(1):356–64. doi: 10.1080/22221751.2021.1888658 (PMC7928038; doi:10.1080/22221751.2021.1888658)
Supplement: Supplemental_material_for_review_.docx [file TEMI_A_1888658_SM6121.docx]

**Figure S1** The dendrogram generated by the *Apa*I enzyme-based pulsed-field gel electrophoresis patterns of the 23 representative *L.innocua* strains was constructed. 12 strains (52.2%) exhibited 6 kinds of identical PTs and STs. The corresponding data, including the name of the strain (Strain ID), PFGE types, serotype, MLST type, the type of sample, sampling date and district, was shown alongside the dendrogram to the right.


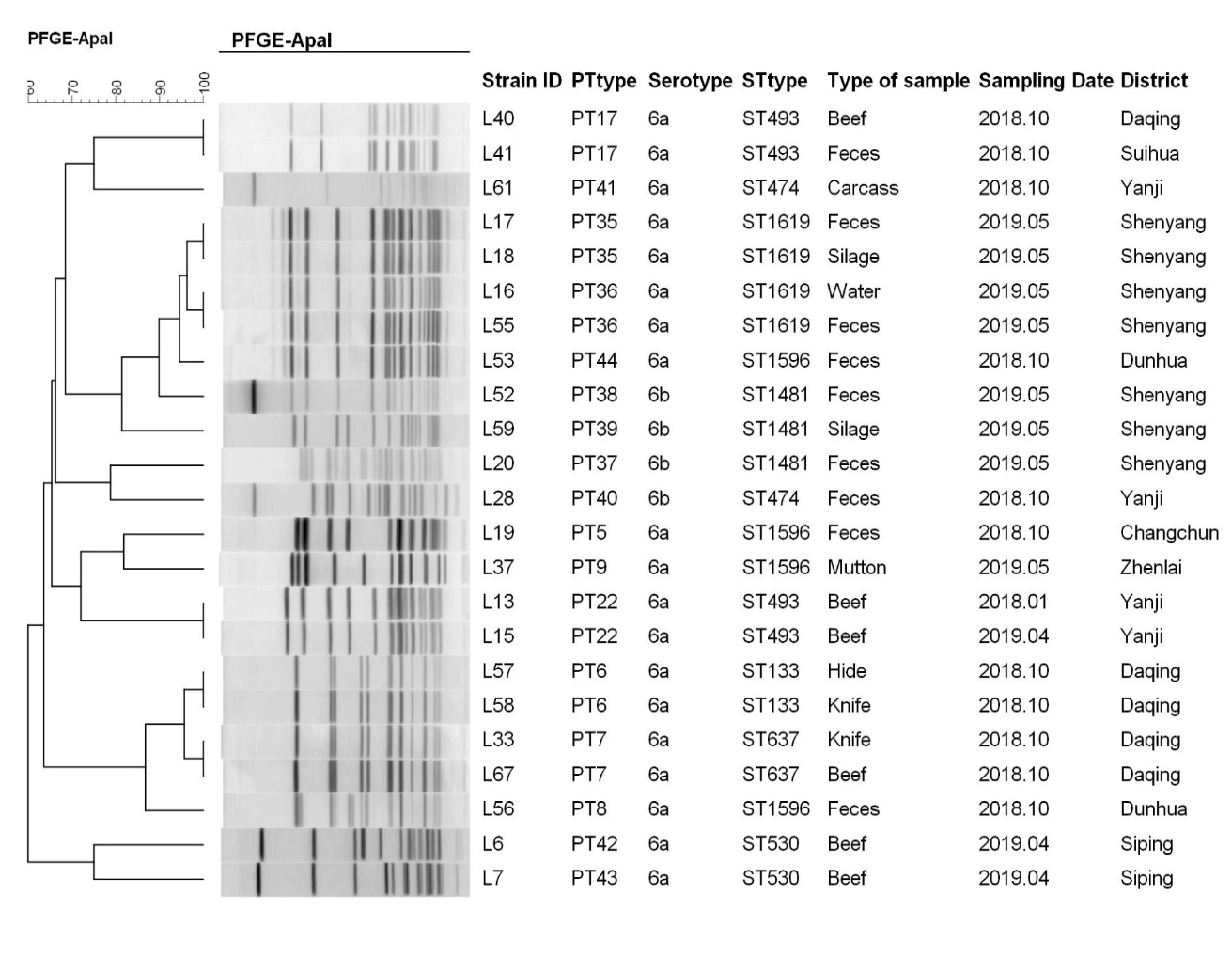


**Figure S2** The minimum spanning tree of the 17 STs of the 50 *L.innocua* isolates from ruminants in farm and slaughtering environments in China. The corresponding sequence type is displayed around the circles. The size of each circle corresponds to the isolate count, and the color within the circles represent the type of region (A) or sample (B).


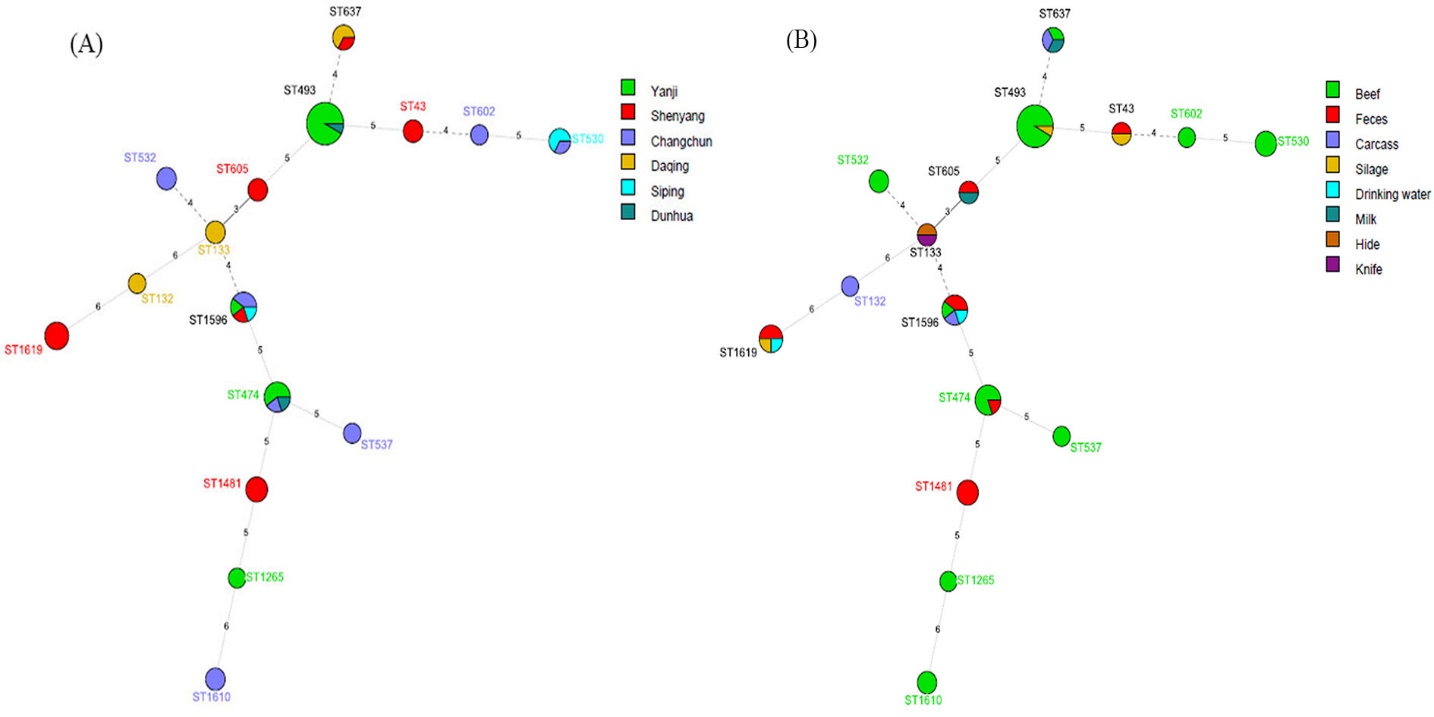


| Farm | Anal swab | | Hide swab | | Silage | | Drinking water | | Total | |
| --- | --- | --- | --- | --- | --- | --- | --- | --- | --- | --- |
|  | M**^a^** | I**^a^** | M | I | M | I | M | I | M | I |
| A | 0(310) | 1(310) | 0(40) | 0(40) | 0(20) | 0(20) | 0(10) | 0(10) | 0(380) | 1(380) |
| B | 0(110) | 0(110) | 0(20) | 0(20) | 0(11) | 0(11) | 0(9) | 0(9) | 0(150) | 0(150) |
| C | 0(161) | 10(161) | 0(25) | 6(25) | 0(10) | 3(10) | 0(10) | 0(10) | 0(206) | 19(206) |
| D | 0(54) | 0(54) | 0(25) | 0(25) | 0(11) | 0(11) | 0(10) | 0(10) | 0(100) | 0(100) |
| E | 0(336) | 0(336) | 0(30) | 0(30) | 0(22) | 0(22) | 0(12) | 0(12) | 0(400) | 0(400) |
| F | 0(388) | 83(388) | 0(45) | 0(45) | 0(20) | 2(20) | 0(10) | 0(10) | 0(463) | 85(463) |
| G | 0(990) | 27(990) | 0(70) | 0(70) | 0(60) | 1(60) | 0(40) | 0(40) | 0(1160) | 28(1160) |
| H | 7(280) | 58(280) | 0(25) | 0(25) | 2(25) | 7(25) | 1(10) | 4(10) | 10(340) | 69(340) |
| J | 0(130) | 0(130) | —— | —— | —— | —— | —— | —— | 0(130) | 0(130) |
| K | 0(160) | 0(160) | —— | —— | —— | —— | —— | —— | 0(160) | 0(160) |
| L | 1(210) | 9(210) | 0(20) | 0(20) | 0(10) | 1(10) | 0(10) | 0(10) | 1(250) | 10(250) |
| N | 0(155) | 0(155) | 0(20) | 0(20) | 0(15) | 0(15) | 0(10) | 0(10) | 0(200) | 0(200) |
| Total | 8(3284) | 188(3284) | 0(320) | 6(320) | 2(204) | 14(204) | 1(131) | 4(131) | 11(3939) | 212(3939) |
|  | 196(3284) | | 6(320) | | 16(204) | | 5(131) | | 223(3939) | |

**Table S1.** The incidence of *Listeria* species in samples collected from farm environments of cattle and sheep.

**a**: **M**: *L. monocytogenes*; **I**: *L. innocua*.

**Table S2.** The incidence of *Listeria* species in raw milk samples collected from dairy cattle farms in farm environments.

| Farm | Milk catagory | No. of samples Collected | No. (%) of samples positive for *L.m* | No. (%) of samples positive for *L.innocua* | No. (%) of samples positive for *Listeria* spp.(%) |
| --- | --- | --- | --- | --- | --- |
| A | Raw milk | 257 | 9(3.5) | 0(0) | 9(3.5) |
| H | Raw milk | 130 | 0(0) | 2(1.5) | 2(1.5) |
| G | Raw milk | 104 | 0(0) | 0(0) | 0(0) |
| Total | ——— | 491 | 9(1.8) | 2(0.4) | 11(2.2) |

**Table S3.** The incidence of *Listeria* species in samples collected from slaughtering environments of beef cattle and sheep.

| Slaughter-house | Anal swab**^a^** | | Hide swab | | Rinse water | | Knife | | Instrument | | Chopping board | | Carcass | | Meat | | Total | |
| --- | --- | --- | --- | --- | --- | --- | --- | --- | --- | --- | --- | --- | --- | --- | --- | --- | --- | --- |
|  | M**^b^** | I**^b^** | M | I | M | I | M | I | M | I | M | I | M | I | M | I | M | I |
| O | 10(50) | 11(50) | 8(50) | 7(50) | 0(10) | 6(10) | 8(50) | 13(50) | 0(20) | 6(20) | 1(20) | 9(20) | 11(48) | 10(48) | 21(39) | 20(39) | 59(287) | 82(287) |
| P | 0(16) | 0(16) | 0(16) | 10(16) | 0(8) | 4(8) | 0(8) | 4(8) | 0(6) | 4(6) | 0(2) | 2(2) | 0(8) | 4(8) | 0(16) | 12(16) | 0(80) | 40(80) |
| Q | 0(10) | 0(10) | 0(10) | 4(10) | 0(5) | 2(5) | 0(6) | 0(6) | 0(4) | 0(4) | 0(10) | 4(10) | 0(15) | 3(15) | 0(10) | 5(10) | 0(70) | 18(70) |
| R | — | —— | — | —— | — | ­—— | — | —— | — | —— | — | —— | — | —— | 0(20) | 6(20) | 0(20) | 6(20) |
| S | — | —— | — | —— | — | —— | — | —— | — | —— | — | —— | — | —— | 10(174) | 118(174) | 10(174) | 118(174)^c^ |
| T | 0(45) | 0(45) | — | —— | — | —— | — | —— | — | —— | — | —— | — | —— | 5(108) | 30(108) | 5(153) | 30(153) |
| Total | 10(121) | 11(121) | 8(76) | 21(76) | 0(23) | 12(23) | 8(64) | 17(64) | 0(30) | 10(30) | 1(32) | 15(32) | 11(71) | 17(71) | 36(367) | 191(367) | 74(784) | 294(784) |
|  | 21(121) | | 29(76) | | 12(23) | | 25(64) | | 10(30) | | 16(32) | | 28(71) | | 220(367) | | 361(784)**^c^** | |

**a**: Anal swabs were got from beef cattle in relevant farms in breeding environment.

**b**: **M**: *L. monocytogenes*; **I**: *L. innocua*.

**c**: *L. monocytogenes* and *L. innocua* coexisted in seven beef samples. Therefore, the total number of *Listeria* positive samples was not 368 but was 361.

| Sampling date | Number of sample | M**^a^**(%) | I**^a^**(%) | *Listeria* (%) |
| --- | --- | --- | --- | --- |
| 2018.08.24 | 10 | 0(0) | 0(0) | 0(0) |
| 2018.10.25 | 13 | 2(15.4) | 9(46.2) | 11(84.6) |
| 2019.01.25 | 13 | 8(61.5) | 10(49.2) | 11(84.6)^b^ |
| 2020.07.23 | 13 | 0(0) | 5(38.5) | 5(38.5) |
| 2020.08.06 | 30 | 0(0) | 27(90) | 27(90) |
| 2020.08.11 | 17 | 0(0) | 1(5.88) | 1(5.88) |
| 2020.08.17 | 26 | 0(0) | 20(76.9) | 20(76.9) |
| 2020.09.17 | 22 | 0(0) | 18(81.8) | 18(81.8) |
| 2020.09.21 | 30 | 0(0) | 28(93.3) | 28(93.3) |
| Total | 174 | 10(5.7) | 118(67.8) | 121(69.5)**^b^** |

**Table S4**. The incidence of *Listeria* in samples collected from abattoir S in Changchun in different batches.

**a**: **M**: *L. monocytogenes*; **I**: *L. innocua*.

**b**: *L. monocytogenes* and *L. innocua* coexisted in 7 beef samples. Therefore, the total number of *Listeria* positive samples was not 128 but was 121.

**Table S5**. The incidence of *Listeria* in samples collected from abattoir T in Yanji in different batches.

| Sampling date | Number of sample | M**^a^**(%) | I**^a^**(%) | *Listeria* (%) |
| --- | --- | --- | --- | --- |
| 2018.10.15 | 29 | 1 (3.4) | 5(17.2) | 6(65.5) |
| 2019.04.15 | 33 | 1(3.0) | 6(18.2) | 7(24.2) |
| 2019.10.15 | 30 | 2(6.7) | 15(50) | 17(56.7) |
| 2020.03.15 | 61 | 1(1.6) | 4(6.6) | 5(8.2) |
| Total | 153 | 5(3.3) | 30(19.6) | 35(22.9) |

**a**: **M**: *L. monocytogenes*; **I**: *L. innocua*.

**Table S6**. The quantitative level of *Listeria* spp. contamination in the four abattoirs.

| *Listeria* count CFU/g or ml | | | | | | |
| --- | --- | --- | --- | --- | --- | --- |
| ***Listeria*** | <10 | 10-10^2^ | 10^2^-10^3^ | 10^3^-10^4^ | 10^4^-10^5^ | Total |
| Anal swab |  | 3 | 2 | 1 |  | 6 |
| Fur swab | 3 | 6 |  |  |  | 9 |
| Rinse water |  | 6 |  |  |  | 6 |
| Knife | 1 | 4 | 5 | 3 |  | 13 |
| Instrument |  | 3 | 3 |  |  | 6 |
| Chopping board | 3 | 3 | 4 | 3 |  | 13 |
| Carcass | 1 | 5 | 6 |  |  | 12 |
| Mutton | 1 | 3 | 2 | 1 | 1 | 8 |
| Beef | 2 | 5 | 5 | 8 | 1 | 21 |
| Total | 11 | 38 | 27 | 16 | 2 | 94**^a^** |

**a**: The total number of plate-counted *Listeria* positive samples was not equal to that of the actual *Listeria* positive samples. This is probably because the direct plate counting method with original samples had no primary enrichment.
